# Arch vessel injury: geometrical considerations. Implications for the mechanism of traumatic myocardial infarction II

**DOI:** 10.1186/1749-7922-1-28

**Published:** 2006-09-08

**Authors:** Rovshan M Ismailov

**Affiliations:** 1 Department of Epidemiology, Graduate School of Public Health, University of Pittsburgh, Pittsburgh, PA 15213, USA

## Abstract

**Background:**

Various types of vascular injury have been reported in the medical literature; the isthmic part of the aorta is at particularly high risk of traumatic rupture. Early diagnosis results in better survival, justifying the search for potential risk factors and diagnostic tests. The aim of this research was to investigate the complex mechanism of blunt injury to the vascular wall with particular focus on the branching region of the vessels. Geometric peculiarities were investigated.

**Methods:**

Multi-phase equations have been used. The system of equations with certain boundary conditions was solved numerically by applying the finite-difference method with order of approximation equal to 0.0001.

**Results:**

The degree of curvature (the Dean number) is highly informative about the shear stress on the external surface of the vessel. An important function of the blood flow on the external wall is to destroy rouleaux. The viscosity of phase 2 (*f*_2_) exceeds, by many times, the viscosity of phase 1 (*f*_1_). The major stress created by blood flow is expressed as the shear stress of *f*_2_. The volume fraction of rouleaux depends to a greater degree on the concentration of erythrocytes (expressed as the viscosity of the mixture) than on the shear stress. The peculiarities of rouleaux formation were assessed and their impact on the local shear stress and, therefore, on the internal wall was determined in relation to the erythrocyte concentration.

**Conclusion:**

The results of this research take into account certain geometrical peculiarities of the branching part of the vessel. The mathematical model created in this study will improve our understanding of the complex mechanism of blunt injury to the vascular wall and, therefore, conditions such as aortic rupture and traumatic acute myocardial infarction.

## Background

Arterial lesions are widely recognized outcomes in trauma patients. In the USA, approximately 7,500 to 8,000 cases of blunt aortic injury occur each year, of which only about 1,000 to 1,500 survive [1-4]. Blunt aortic injuries are responsible for up to 40% of fatalities occurring in traffic accidents [5-7]. Studies based on autopsy findings have shown that between 12 and 29% of all traffic fatalities have additional thoracic aortic traumas [2-4,7,8]. In patients with multiple injuries, the incidence of blunt thoracic aortic injury ranges from 3 to 17% [2,8-11]. In a study by Smith et al. [1], blunt trauma to the aorta was found to be the second most common cause of death following head injury.

The main causes of blunt traumatic aortic injuries (76%) are lateral and head-on motor vehicle collisions at speeds greater than 50 km/h, or accidents associated with substantial car deformation, followed by falls from heights and crush injuries [12,13]. Coronary dissection and rupture resulting from trauma have also been reported [14-18] where blunt injury was found to be the leading mechanism [19-21].

The isthmic part of the vessel was found to be at particularly high risk of rupture [22]. Laceration or rupture of the aortic isthmus has been previously reported in the medical literature [23]. Several mechanisms have been proposed to explain traumatized arterial bifurcations. Deceleration forces exerted on the aortic branches as the result of a frontal collision can lead to the rupture of the isthmus [24,25]. Compression of the aorta between the spine and thorax has been shown to cause isthmic lacerations [26]. Finally, torsion, shearing and bending forces are exerted on the isthmus, leading to its rupture during frontal and side motor vehicle impacts when rapid deceleration and chest compression are combined [23].

As we showed previously, blunt trauma may lead to certain hemodynamic peculiarities that can cause damage to the endothelium and rupture of the vessel [21,27]. The aim of this research was to investigate further the complex mechanism of blunt injury to the vascular wall, with particular focus on branching parts of the vessels. Geometric and rheological peculiarities were investigated.

## Methods and results

### A. External wall: considerations for curvature, shear stress and blood flow velocity

When blood flows through the branching area in the aorta and large arteries it changes direction. When a fluid runs through tubes a change of the fluid direction also occurs, and this change is evaluated in terms of the centrifugal force that acts on particles toward the external rounding off. This action results in a secondary flow, and redistribution of velocities takes place. On the external wall, the velocity of flow and the shear stress increase, but they decrease on the internal surface of the wall [28,29]. The results of experimental studies showed that the increase in the shear stress and the influence of the curvature of the vessel on resistance are considerably higher during laminar flow than turbulent flow [28,29].

Assuming that blood flow in the cardiovascular system is predominantly laminar [29], let me consider the shear stress within the laminar flow on the external wall of the branching part of the vessel. It is known [29] that branching regions of arteries have various angles. In general, for their description, the coefficient of branching out is applied (i.e. the relationship among sum of areas, angles of branching, divisor of flow, profiles, velocities, Reynolds number, radius of curvature of internal wall at branching, diameters of diverging vessels) [29]. Various approaches to evaluate the degree of branching of the aorta make the classification of branching difficult.

**Figure 1:**
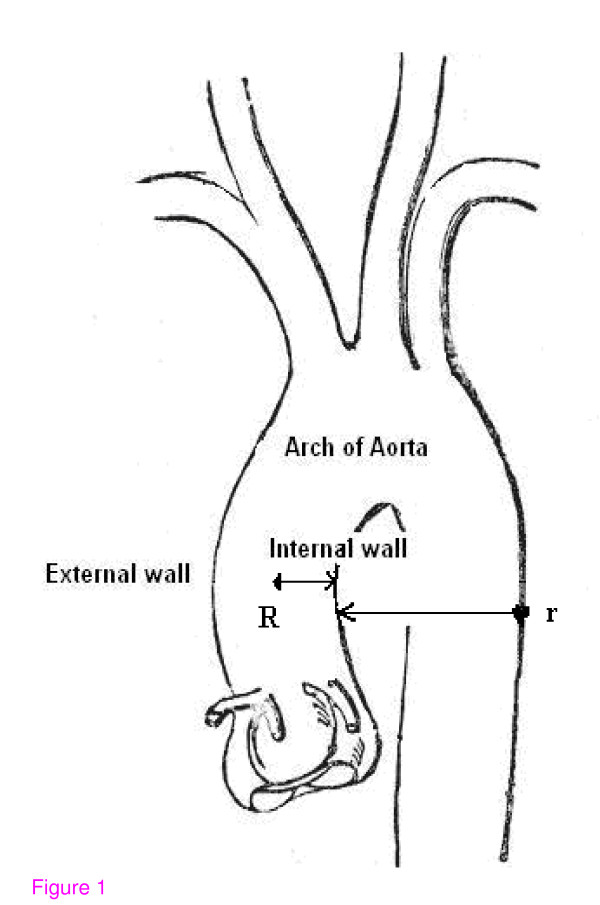
Arch of aorta. External, internal vessel walls and the values of curvature (R and r).

As in previous research [28], let me take the Dean number as a parameter to determine the influence of curvature on the resistance to blood flow. I am interested in considering the following Reynolds number range that most closely corresponds to human (physiological) range:

101.6<Re⁡((Rr)1/2)<103
 MathType@MTEF@5@5@+=feaafiart1ev1aaatCvAUfKttLearuWrP9MDH5MBPbIqV92AaeXatLxBI9gBaebbnrfifHhDYfgasaacH8akY=wiFfYdH8Gipec8Eeeu0xXdbba9frFj0=OqFfea0dXdd9vqai=hGuQ8kuc9pgc9s8qqaq=dirpe0xb9q8qiLsFr0=vr0=vr0dc8meaabaqaciaacaGaaeqabaqabeGadaaakeaacqaIXaqmcqaIWaamcqaIXaqmcqGGUaGlcqaI2aGncqGH8aapcyGGsbGucqGGLbqzcqGGOaakcqGGOaakdaWcaaqaaiabdkfasbqaaiabdkhaYbaacqGGPaqkdaahaaWcbeqaaiabigdaXiabc+caViabikdaYaaakiabcMcaPiabgYda8iabigdaXiabicdaWmaaCaaaleqabaGaeG4mamdaaaaa@41EC@

where R = radius of the tube (vessel), r = radius of the curvature that can be applied to various branching areas of the entire cardiovascular system [29]. Let me show how the Dean number describes conditions arising from various anatomical variations of the canine aorta. For this I shall take three average curvature values that correspond closely to the curvature of the aorta in general: R = 7 mm, r = 25 mm; the maximal value of curvature, R = 7 mm, r = 15 mm; the minimal value of curvature of the ascending aorta, R = 7 mm, r = 40 mm (Figure 1).

If flow is laminar, then the Dean number is determined as:

D=0.5 Re⁡((Rr)1/2)
 MathType@MTEF@5@5@+=feaafiart1ev1aaatCvAUfKttLearuWrP9MDH5MBPbIqV92AaeXatLxBI9gBaebbnrfifHhDYfgasaacH8akY=wiFfYdH8Gipec8Eeeu0xXdbba9frFj0=OqFfea0dXdd9vqai=hGuQ8kuc9pgc9s8qqaq=dirpe0xb9q8qiLsFr0=vr0=vr0dc8meaabaqaciaacaGaaeqabaqabeGadaaakeaacqqGebarcqGH9aqpcqaIWaamcqGGUaGlcqaI1aqncqqGGaaicyGGsbGucqGGLbqzcqGGOaakcqGGOaakdaWcaaqaaiabdkfasbqaaiabdkhaYbaacqGGPaqkdaahaaWcbeqaaiabigdaXiabc+caViabikdaYaaakiabcMcaPaaa@3DDF@

where Re = Re⁡=(dρU∞)μ
 MathType@MTEF@5@5@+=feaafiart1ev1aaatCvAUfKttLearuWrP9MDH5MBPbIqV92AaeXatLxBI9gBaebbnrfifHhDYfgasaacH8akY=wiFfYdH8Gipec8Eeeu0xXdbba9frFj0=OqFfea0dXdd9vqai=hGuQ8kuc9pgc9s8qqaq=dirpe0xb9q8qiLsFr0=vr0=vr0dc8meaabaqaciaacaGaaeqabaqabeGadaaakeaacyGGsbGucqGGLbqzcqGH9aqpdaWcaaqaamaabmGabaGaemizaqgcciGae8xWdiNaemyvau1aaSbaaSqaaiabg6HiLcqabaaakiaawIcacaGLPaaaaeaacqWF8oqBaaaaaa@3970@

and *d *= diameter of the vessel, *μ *= fluid viscosity, *ρ *= density and *U*_∞ _= blood flow velocity.

The value of the coefficient of resistance (*λ*) is calculated by the following formula [28]:

(λλ0)=0.37 D0.36
 MathType@MTEF@5@5@+=feaafiart1ev1aaatCvAUfKttLearuWrP9MDH5MBPbIqV92AaeXatLxBI9gBaebbnrfifHhDYfgasaacH8akY=wiFfYdH8Gipec8Eeeu0xXdbba9frFj0=OqFfea0dXdd9vqai=hGuQ8kuc9pgc9s8qqaq=dirpe0xb9q8qiLsFr0=vr0=vr0dc8meaabaqaciaacaGaaeqabaqabeGadaaakeaacqGGOaakdaWcaaqaaGGaciab=T7aSbqaaiab=T7aSnaaBaaaleaacqaIWaamaeqaaaaakiabcMcaPiabg2da9iabicdaWiabc6caUiabiodaZiabiEda3iabbccaGiabbseaenaaCaaaleqabaGaeGimaaJaeiOla4IaeG4mamJaeGOnaydaaaaa@3D87@

where *λ*_0 _= the coefficient of resistance of a straight tube by the formula [28]:

(1λ0)=2.0 lg(udλ0v)−0.8     (1)
 MathType@MTEF@5@5@+=feaafiart1ev1aaatCvAUfKttLearuWrP9MDH5MBPbIqV92AaeXatLxBI9gBaebbnrfifHhDYfgasaacH8akY=wiFfYdH8Gipec8Eeeu0xXdbba9frFj0=OqFfea0dXdd9vqai=hGuQ8kuc9pgc9s8qqaq=dirpe0xb9q8qiLsFr0=vr0=vr0dc8meaabaqaciaacaGaaeqabaqabeGadaaakeaacqGGOaakdaWcaaqaaiabigdaXaqaamaakaaabaacciGae83UdW2aaSbaaSqaaiabicdaWaqabaaabeaaaaGccqGGPaqkcqGH9aqpcqaIYaGmcqGGUaGlcqaIWaamcqqGGaaicqqGSbaBcqqGNbWzcqGGOaakdaWcaaqaaiabdwha1jabdsgaKnaakaaabaGae83UdW2aaSbaaSqaaiabicdaWaqabaaabeaaaOqaaGqaciab+zha2baacqGGPaqkcqGHsislcqaIWaamcqGGUaGlcqaI4aaocaWLjaGaaCzcamaabmGabaGaeGymaedacaGLOaGaayzkaaaaaa@49F2@

where *v *= kinematic viscosity [28].

The shear stress is determined by the formula [29]:

τ=λρu28     (2)
 MathType@MTEF@5@5@+=feaafiart1ev1aaatCvAUfKttLearuWrP9MDH5MBPbIqV92AaeXatLxBI9gBaebbnrfifHhDYfgasaacH8akY=wiFfYdH8Gipec8Eeeu0xXdbba9frFj0=OqFfea0dXdd9vqai=hGuQ8kuc9pgc9s8qqaq=dirpe0xb9q8qiLsFr0=vr0=vr0dc8meaabaqaciaacaGaaeqabaqabeGadaaakeaaiiGacqWFepaDcqGH9aqpdaWcaaqaaiab=T7aSjab=f8aYjabdwha1naaCaaaleqabaGaeGOmaidaaaGcbaGaeGioaGdaaiaaxMaacaWLjaWaaeWaceaacqaIYaGmaiaawIcacaGLPaaaaaa@3A53@

Where *u *is the average flow velocity.

Calculations for the three modes of curvature are given in Tables 1, 2 and 3.

**Table 1:** Average value of curvature (Rr)1/2
 MathType@MTEF@5@5@+=feaafiart1ev1aaatCvAUfKttLearuWrP9MDH5MBPbIqV92AaeXatLxBI9gBaebbnrfifHhDYfgasaacH8akY=wiFfYdH8Gipec8Eeeu0xXdbba9frFj0=OqFfea0dXdd9vqai=hGuQ8kuc9pgc9s8qqaq=dirpe0xb9q8qiLsFr0=vr0=vr0dc8meaabaqaciaacaGaaeqabaqabeGadaaakeaacqGGOaakdaWcaaqaaiabdkfasbqaaiabdkhaYbaacqGGPaqkdaahaaWcbeqaaiabigdaXiabc+caViabikdaYaaaaaa@33FD@ = 0.52.

**Velocity (m/s)**	**Re number**	**The Dean number**	λ	**Shear stress, τ 10^3 ^(N/m^2^)**
0.2	747	194	0,14	0.707
0.4	1494	388.4	0.189	3.81
0.6	2241	582.6	0.21	9.54
1	3735	971.1	0.26	32.8
1.2	4482	1165.3	0.281	50.9

**Table 2:** Average value of curvature (Rr)1/2
 MathType@MTEF@5@5@+=feaafiart1ev1aaatCvAUfKttLearuWrP9MDH5MBPbIqV92AaeXatLxBI9gBaebbnrfifHhDYfgasaacH8akY=wiFfYdH8Gipec8Eeeu0xXdbba9frFj0=OqFfea0dXdd9vqai=hGuQ8kuc9pgc9s8qqaq=dirpe0xb9q8qiLsFr0=vr0=vr0dc8meaabaqaciaacaGaaeqabaqabeGadaaakeaacqGGOaakdaWcaaqaaiabdkfasbqaaiabdkhaYbaacqGGPaqkdaahaaWcbeqaaiabigdaXiabc+caViabikdaYaaaaaa@33FD@ = 0.68.

**Velocity (m/s)**	**Re number**	**The Dean number**	λ	**Shear stress, τ 10^3 ^(N/m^2^)**
0.2	747	253	0,16	0.8
0.4	1494	507	0.21	4.2
0.6	2241	762	0.24	10.92
1	3735	1270	0.29	36.61
1.2	4482	1524	0.31	56.35

**Table 3:** Average value of curvature (Rr)1/2
 MathType@MTEF@5@5@+=feaafiart1ev1aaatCvAUfKttLearuWrP9MDH5MBPbIqV92AaeXatLxBI9gBaebbnrfifHhDYfgasaacH8akY=wiFfYdH8Gipec8Eeeu0xXdbba9frFj0=OqFfea0dXdd9vqai=hGuQ8kuc9pgc9s8qqaq=dirpe0xb9q8qiLsFr0=vr0=vr0dc8meaabaqaciaacaGaaeqabaqabeGadaaakeaacqGGOaakdaWcaaqaaiabdkfasbqaaiabdkhaYbaacqGGPaqkdaahaaWcbeqaaiabigdaXiabc+caViabikdaYaaaaaa@33FD@ = 0.41.

**Velocity (m/s)**	**Re number**	**The Dean number**	λ	**Shear stress, τ 10^3 ^(N/m^2^)**
0.2	747	153	0.13	0.65
0.4	1494	306	0.17	3.43
0.6	2241	459	0.2	9.1
1	3735	765.6	0.24	30.3
1.2	4482	918.8	0.26	47.2

According to these tables, the degree of curvature (the Dean number) is highly informative with regard to the shear stress on the external surface of the vessel. For instance, for the ascending aorta with an average blood velocity of 0.2 m/s, the shear stress is 1.5–2 times greater than that calculated for a straight (plane) tube (*τ *= 0.43 N/m^2^) [29]. Also, according to tables 1, 2, 3, the curvature may play a significant role when the vessel is compressed as the result, for example, of injury. The compressed part of the vessel can increase the shear stress [27]; however, when the curvature is significant, the shear stress may become even greater owing to the influence of both curvature and compression. Thus, the Dean number is an important factor to consider when determining the shear stress acting on the external wall of the vessel. In addition, according to tables 1, 2, 3, the shear stress may exceed the physiological threshold calculated for the endothelium (i.e. 40 N/m^2^) at the extreme value of blood velocity (1.2 m/s) [27,29].

According to the above and to our previous research [21,27], three internal factors have been identified (the Dean number, compression and blood flow velocity) that can play a dominant role with respect to endothelium damages and resulting vessel rupture. Let me now investigate the conditions that appear as the result of fluid (plasma) and particle (erythrocyte) movement, i.e. the multiphase character of the medium. It will allow me to look at the role of erythrocyte concentration, blood viscosity and rouleaux formation on the external surface of the vessel.

### B. External wall: additional considerations for blood viscosity, erythrocyte concentration and rouleaux formation

Let me now determine the association between yield velocity and shear stress [27,29]. Calculations are given in Tables 4, 5 and 6.

**Table 4:** The relationship between blood viscosity, shear stress and yield velocity at concentration of erythrocytes 28.7%.

**Blood viscosity (mNcm^-2^)**	**Shear stress, τ 10^3 ^(N/m**^2^)	**Yield velocity (m^-1^)**
12	2.4	0.2
11	5.5	0.5
8	8	1
5	25	5
4	40	10
3	200	50

**Table 5:** The relationship between blood viscosity, shear stress and yield velocity at concentration of erythrocytes 35.9%.

**Blood viscosity (mNcm^-2^)**	**Shear stress, τ 10^3 ^(N/m**^2^)	**Yield velocity (m^-1^)**
29	5.8	0.2
18	9	0.5
14	14	1
8	40	5
7	70	10
5	250	50

**Table 6:** The relationship between the blood viscosity, shear stress and yield velocity at concentration of erythrocytes 48%.

**Blood viscosity (mNcm^-2^)**	**Shear stress, τ 10^3 ^(N/m^2^)**	**Yield velocity (m^-1^)**
60	12	0.2
38	19	0.5
29	29	1
14	70	5
11	110	10
9	450	50

According to tables 4, 5, 6, shear stress exceeds the maximal physiological value (40 N/m2) when blood viscosity is around 3 mNcm^-2 ^and the concentration of erythrocytes 28.7 %. Alternatively, at high yield velocity (10 – 50 m^-1^), only a high concentration of erythrocytes (48% and higher) can result in abnormal values of blood viscosity [29].

When the blood flow velocity is moderate (i.e. 0.1 – 0.4 m/s) [29] and the shear stress calculated by the formulae (1, 2) equals 0.15 mNm^-2 ^(i.e. physiological value), then according to table 6 it is possible that the viscosity will increase up to 9–11 mNm^-2 ^if the erythrocyte concentration equals 48% or more and the yield velocity is within the range 10 to 50 c^-1 ^(calculations are made at blood flow rate = 0.1 m/s) Therefore, it can be concluded that only quite specific conditions such as an increase in the concentration of erythrocytes (48% or more) and relatively slow motion of blood (equal to or less than 0.1 mc^-1^) [21] may lead to the formation of rouleaux and increase the shear stress on the external vessel wall.

In addition, according to tables 4, 5, 6, it can be seen that the shear stress values are different at equal yield velocities. This indicates that two phases participate in creating the shear stress, since if the flow of blood were homogeneous the stress would be the same.

For two-phase flow, the total shear stress is the sum of the shear stresses of the two phases considered separately:

τ=μ1f1(∂u1∂y1)+μ2f2(∂u2∂y2)     (3)
 MathType@MTEF@5@5@+=feaafiart1ev1aaatCvAUfKttLearuWrP9MDH5MBPbIqV92AaeXatLxBI9gBaebbnrfifHhDYfgasaacH8akY=wiFfYdH8Gipec8Eeeu0xXdbba9frFj0=OqFfea0dXdd9vqai=hGuQ8kuc9pgc9s8qqaq=dirpe0xb9q8qiLsFr0=vr0=vr0dc8meaabaqaciaacaGaaeqabaqabeGadaaakeaaiiGacqWFepaDcqGH9aqpcqWF8oqBdaWgaaWcbaGaeGymaedabeaakiabdAgaMnaaBaaaleaacqaIXaqmaeqaaOGaeiikaGYaaSaaaeaacqWFciITcqWG1bqDdaWgaaWcbaGaeGymaedabeaaaOqaaiab=jGi2kabdMha5naaBaaaleaacqaIXaqmaeqaaaaakiabcMcaPiabgUcaRiab=X7aTnaaBaaaleaacqaIYaGmaeqaaOGaemOzay2aaSbaaSqaaiabikdaYaqabaGccqGGOaakdaWcaaqaaiab=jGi2kabdwha1naaBaaaleaacqaIYaGmaeqaaaGcbaGae8NaIyRaemyEaK3aaSbaaSqaaiabikdaYaqabaaaaOGaeiykaKIaaCzcaiaaxMaadaqadiqaaiabiodaZaGaayjkaiaawMcaaaaa@5243@

where phase 1 (*f*_1_) is liquid plasma and phase 2 (*f*_2_) is a relatively solid phase (erythrocytes and rouleaux). From this point of view, let me consider the theory of a multi-phase medium [21,27,30], and in particular the relationship between the longitudinal and transverse velocities of the phases in the boundary layer during flow around a flat surface [27].

Let us assume that the carrying flow, in a direction parallel to the flat surface of the vessel, is viscous, and the flow itself (solid particles = erythrocytes) is ideal. Also, we will assume that axis x is along the direction of flow while axis y is perpendicular to the surface. Then the two equations for plasma (1^st ^phase) will take the following form:

ρ1u1∂u1∂x+ρ1v1∂u1∂y=μ∂2u1∂y2+k(u2−u1),     (4)
 MathType@MTEF@5@5@+=feaafiart1ev1aaatCvAUfKttLearuWrP9MDH5MBPbIqV92AaeXatLxBI9gBaebbnrfifHhDYfgasaacH8akY=wiFfYdH8Gipec8Eeeu0xXdbba9frFj0=OqFfea0dXdd9vqai=hGuQ8kuc9pgc9s8qqaq=dirpe0xb9q8qiLsFr0=vr0=vr0dc8meaabaqaciaacaGaaeqabaqabeGadaaakeaaiiGacqWFbpGCdaWgaaWcbaGaeGymaedabeaakiabdwha1naaBaaaleaacqaIXaqmaeqaaOWaaSaaaeaacqWFciITcqWG1bqDdaWgaaWcbaGaeGymaedabeaaaOqaaiab=jGi2kabdIha4baacqGHRaWkcqWFbpGCdaWgaaWcbaGaeGymaedabeaakiabdAha2naaBaaaleaacqaIXaqmaeqaaOWaaSaaaeaacqWFciITcqWG1bqDdaWgaaWcbaGaeGymaedabeaaaOqaaiab=jGi2kabdMha5baacqGH9aqpcqWF8oqBdaWcaaqaaiab=jGi2oaaCaaaleqabaGaeGOmaidaaOGaemyDau3aaSbaaSqaaiabigdaXaqabaaakeaacqWFciITcqWG5bqEdaahaaWcbeqaaiabikdaYaaaaaGccqGHRaWkcqWGRbWAcqGGOaakcqWG1bqDdaWgaaWcbaGaeGOmaidabeaakiabgkHiTiabdwha1naaBaaaleaacqaIXaqmaeqaaOGaeiykaKIaeiilaWIaaCzcaiaaxMaadaqadiqaaiabisda0aGaayjkaiaawMcaaaaa@60F6@

∂(ρ1u1)∂x+∂(ρ1v1)∂y=0.     (5)
 MathType@MTEF@5@5@+=feaafiart1ev1aaatCvAUfKttLearuWrP9MDH5MBPbIqV92AaeXatLxBI9gBaebbnrfifHhDYfgasaacH8akY=wiFfYdH8Gipec8Eeeu0xXdbba9frFj0=OqFfea0dXdd9vqai=hGuQ8kuc9pgc9s8qqaq=dirpe0xb9q8qiLsFr0=vr0=vr0dc8meaabaqaciaacaGaaeqabaqabeGadaaakeaadaWcaaqaaGGaciab=jGi2kabcIcaOiab=f8aYnaaBaaaleaacqaIXaqmaeqaaOGaemyDau3aaSbaaSqaaiabigdaXaqabaGccqGGPaqkaeaacqWFciITcqWG4baEaaGaey4kaSYaaSaaaeaacqWFciITcqGGOaakcqWFbpGCdaWgaaWcbaGaeGymaedabeaakiabdAha2naaBaaaleaacqaIXaqmaeqaaOGaeiykaKcabaGae8NaIyRaemyEaKhaaiabg2da9iabicdaWiabc6caUiaaxMaacaWLjaWaaeWaceaacqaI1aqnaiaawIcacaGLPaaaaaa@4B23@

where *κ *is a coefficient of phase interaction [31]. The three equations for erythrocytes (2^nd ^phase) will take the following form:

ρ2u2∂u2∂x+ρ2v2∂u2∂y=k(u2−u1),     (6)
 MathType@MTEF@5@5@+=feaafiart1ev1aaatCvAUfKttLearuWrP9MDH5MBPbIqV92AaeXatLxBI9gBaebbnrfifHhDYfgasaacH8akY=wiFfYdH8Gipec8Eeeu0xXdbba9frFj0=OqFfea0dXdd9vqai=hGuQ8kuc9pgc9s8qqaq=dirpe0xb9q8qiLsFr0=vr0=vr0dc8meaabaqaciaacaGaaeqabaqabeGadaaakeaaiiGacqWFbpGCdaWgaaWcbaGaeGOmaidabeaakiabdwha1naaBaaaleaacqaIYaGmaeqaaOWaaSaaaeaacqWFciITcqWG1bqDdaWgaaWcbaGaeGOmaidabeaaaOqaaiab=jGi2kabdIha4baacqGHRaWkcqWFbpGCdaWgaaWcbaGaeGOmaidabeaakiabdAha2naaBaaaleaacqaIYaGmaeqaaOWaaSaaaeaacqWFciITcqWG1bqDdaWgaaWcbaGaeGOmaidabeaaaOqaaiab=jGi2kabdMha5baacqGH9aqpcqWGRbWAcqGGOaakcqWG1bqDdaWgaaWcbaGaeGOmaidabeaakiabgkHiTiabdwha1naaBaaaleaacqaIXaqmaeqaaOGaeiykaKIaeiilaWIaaCzcaiaaxMaadaqadiqaaiabiAda2aGaayjkaiaawMcaaaaa@553F@

ρ2u2∂v2∂x+ρ2v2∂v2∂y=k(v2−v1),     (7)
 MathType@MTEF@5@5@+=feaafiart1ev1aaatCvAUfKttLearuWrP9MDH5MBPbIqV92AaeXatLxBI9gBaebbnrfifHhDYfgasaacH8akY=wiFfYdH8Gipec8Eeeu0xXdbba9frFj0=OqFfea0dXdd9vqai=hGuQ8kuc9pgc9s8qqaq=dirpe0xb9q8qiLsFr0=vr0=vr0dc8meaabaqaciaacaGaaeqabaqabeGadaaakeaaiiGacqWFbpGCdaWgaaWcbaGaeGOmaidabeaakiabdwha1naaBaaaleaacqaIYaGmaeqaaOWaaSaaaeaacqWFciITcqWG2bGDdaWgaaWcbaGaeGOmaidabeaaaOqaaiab=jGi2kabdIha4baacqGHRaWkcqWFbpGCdaWgaaWcbaGaeGOmaidabeaaieGakiab+zha2naaBaaaleaacqaIYaGmaeqaaOWaaSaaaeaacqWFciITcqWG2bGDdaWgaaWcbaGaeGOmaidabeaaaOqaaiab=jGi2kabdMha5baacqGH9aqpcqWGRbWAcqGGOaakcqWG2bGDdaWgaaWcbaGaeGOmaidabeaakiabgkHiTiabdAha2naaBaaaleaacqaIXaqmaeqaaOGaeiykaKIaeiilaWIaaCzcaiaaxMaadaqadiqaaiabiEda3aGaayjkaiaawMcaaaaa@554F@

∂(ρ2u2)∂x+∂(ρ2v2)∂y=0.     (8)
 MathType@MTEF@5@5@+=feaafiart1ev1aaatCvAUfKttLearuWrP9MDH5MBPbIqV92AaeXatLxBI9gBaebbnrfifHhDYfgasaacH8akY=wiFfYdH8Gipec8Eeeu0xXdbba9frFj0=OqFfea0dXdd9vqai=hGuQ8kuc9pgc9s8qqaq=dirpe0xb9q8qiLsFr0=vr0=vr0dc8meaabaqaciaacaGaaeqabaqabeGadaaakeaadaWcaaqaaGGaciab=jGi2kabcIcaOiab=f8aYnaaBaaaleaacqaIYaGmaeqaaOGaemyDau3aaSbaaSqaaiabikdaYaqabaGccqGGPaqkaeaacqWFciITcqWG4baEaaGaey4kaSYaaSaaaeaacqWFciITcqGGOaakcqWFbpGCdaWgaaWcbaGaeGOmaidabeaakiabdAha2naaBaaaleaacqaIYaGmaeqaaOGaeiykaKcabaGae8NaIyRaemyEaKhaaiabg2da9iabicdaWiabc6caUiaaxMaacaWLjaWaaeWaceaacqaI4aaoaiaawIcacaGLPaaaaaa@4B31@

An equation for the 1^st ^and 2^nd ^phases will take the following form:

The boundary conditions for the system of partial differential equations (4)-(9) depend on *x *and *y*. Thus, for *x *= *x*_0 _and any *y*, I have:

ρ2ρ20+ρ1ρ10=1.     (9)
 MathType@MTEF@5@5@+=feaafiart1ev1aaatCvAUfKttLearuWrP9MDH5MBPbIqV92AaeXatLxBI9gBaebbnrfifHhDYfgasaacH8akY=wiFfYdH8Gipec8Eeeu0xXdbba9frFj0=OqFfea0dXdd9vqai=hGuQ8kuc9pgc9s8qqaq=dirpe0xb9q8qiLsFr0=vr0=vr0dc8meaabaqaciaacaGaaeqabaqabeGadaaakeaadaWcaaqaaGGaciab=f8aYnaaBaaaleaacqaIYaGmaeqaaaGcbaGae8xWdi3aaSbaaSqaaiabikdaYiabicdaWaqabaaaaOGaey4kaSYaaSaaaeaacqWFbpGCdaWgaaWcbaGaeGymaedabeaaaOqaaiab=f8aYnaaBaaaleaacqaIXaqmcqaIWaamaeqaaaaakiabg2da9iabigdaXiabc6caUiaaxMaacaWLjaWaaeWaceaacqaI5aqoaiaawIcacaGLPaaaaaa@41C7@

*u*_1 _= *u*_2 _= *f*_1_, *v*_1 _= *v*_2 _= *f*_2_, *ρ*_2 _= ρ2∗
 MathType@MTEF@5@5@+=feaafiart1ev1aaatCvAUfKttLearuWrP9MDH5MBPbIqV92AaeXatLxBI9gBaebbnrfifHhDYfgasaacH8akY=wiFfYdH8Gipec8Eeeu0xXdbba9frFj0=OqFfea0dXdd9vqai=hGuQ8kuc9pgc9s8qqaq=dirpe0xb9q8qiLsFr0=vr0=vr0dc8meaabaqaciaacaGaaeqabaqabeGadaaakeaaiiGacqWFbpGCdaqhaaWcbaGaeGOmaidabaGaey4fIOcaaaaa@3081@, *ρ*_1 _= ρ1∗
 MathType@MTEF@5@5@+=feaafiart1ev1aaatCvAUfKttLearuWrP9MDH5MBPbIqV92AaeXatLxBI9gBaebbnrfifHhDYfgasaacH8akY=wiFfYdH8Gipec8Eeeu0xXdbba9frFj0=OqFfea0dXdd9vqai=hGuQ8kuc9pgc9s8qqaq=dirpe0xb9q8qiLsFr0=vr0=vr0dc8meaabaqaciaacaGaaeqabaqabeGadaaakeaaiiGacqWFbpGCdaqhaaWcbaGaeGymaedabaGaey4fIOcaaaaa@307F@;     (10)

for *x *> *x*_0 _and y = 0, I have:

*u*_1 _= *u*_2 _= 0, *v*_1 _= 0, *v*_2 _= *f*_3_(*τ*_1_), *ρ*_1 _= 0;     (11)

and for *x *> *x*_0_, *y *= *δ*

*u*_1 _= *u*_2 _= *u*_∞_.     (12)

In the above, *u*_1 _and *u*_2 _are the longitudinal components of velocity; *v*_1 _and *v*_2 _are the transverse components of velocity; *f*_1 _and *f*_2 _= initial distribution of the velocities in the boundary layer; ρ2∗
 MathType@MTEF@5@5@+=feaafiart1ev1aaatCvAUfKttLearuWrP9MDH5MBPbIqV92AaeXatLxBI9gBaebbnrfifHhDYfgasaacH8akY=wiFfYdH8Gipec8Eeeu0xXdbba9frFj0=OqFfea0dXdd9vqai=hGuQ8kuc9pgc9s8qqaq=dirpe0xb9q8qiLsFr0=vr0=vr0dc8meaabaqaciaacaGaaeqabaqabeGadaaakeaaiiGacqWFbpGCdaqhaaWcbaGaeGOmaidabaGaey4fIOcaaaaa@3081@ and ρ1∗
 MathType@MTEF@5@5@+=feaafiart1ev1aaatCvAUfKttLearuWrP9MDH5MBPbIqV92AaeXatLxBI9gBaebbnrfifHhDYfgasaacH8akY=wiFfYdH8Gipec8Eeeu0xXdbba9frFj0=OqFfea0dXdd9vqai=hGuQ8kuc9pgc9s8qqaq=dirpe0xb9q8qiLsFr0=vr0=vr0dc8meaabaqaciaacaGaaeqabaqabeGadaaakeaaiiGacqWFbpGCdaqhaaWcbaGaeGymaedabaGaey4fIOcaaaaa@307F@ = initial distribution of the densities. The beginning of the 2^nd ^phase (due to separation) was determined from the calculated shear stress and the corresponding separation determined from the experimental data.

**Figure 2:**
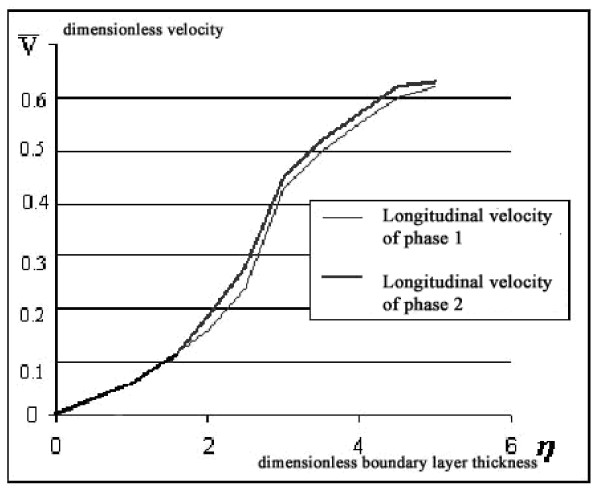
Theoretical distribution of longitudinal velocity.

**Figure 3:**
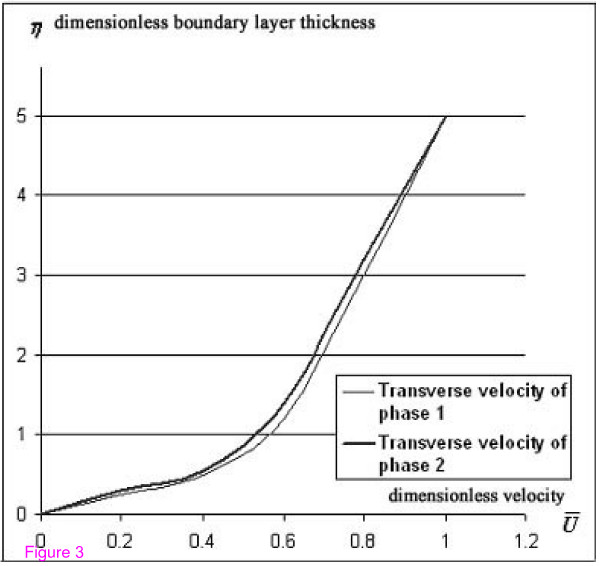
Theoretical distribution of transverse velocity.

The system of equations (4)-(9) with boundary conditions (10)-(12) was solved numerically by applying the finite-difference method with order of approximation equal to 0.0001. For two-phase flow, the shear stress is calculated as a sum of the shear stresses of the two phases. The numerical solution of the system for the equation of the boundary layer for transverse velocities is shown in Figures 2 and 3, where the longitudinal and transverse velocities of the two phases differ negligibly from each other.

This allows me to determine the approximate value of the viscosity of *f*_2 _and the shear stress corresponding to *f*_1 _and *f*_2_. The calculations are given in Tables 7, 8 and 9

**Table 7:** The relationship between yield velocity, whole blood viscosity and shear stress of phases 1 and 2 when erythrocyte concentration is 28%.

**Yield velocity (m^-1^)**	**Blood viscosity μ_2 _(mNcm^-2^)**	**Shear stress corresponding to *f*_1_, τ_1 _10^3 ^(N/m^2^)**	**Shear stress corresponding to *f*_2_, τ_1 _10^3 ^(N/m^2^)**
0.2	39	0.17	2.1
0.5	36.2	0.43	5.06
1	25.5	0.864	7.14
5	14.78	4.32	20.58

**Table 8:** The relationship between yield velocity, whole blood viscosity and shear stress of phases 1 and 2 when erythrocyte concentration is 35.9%.

**Yield velocity (m^-1^)**	**Blood viscosity μ_2 _(mNcm^-2^)**	**Shear stress corresponding to *f*_1_, τ_1 _10^3 ^(N/m^2^)**	**Shear stress corresponding to *f*_2_, τ_1 _10^3 ^(N/m^2^)**
0.2	78.47	0.15	5.6
0.5	47.88	0.38	8.46
1	36.77	0.76	12.9
5	20.88	3.84	36

**Table 9:** The relationship between yield velocity, whole blood viscosity and shear stress of phases 1 and 2 when erythrocyte concentration is 48%.

**Yield velocity (m^-1^)**	**Blood viscosity μ_2 _(mNcm^-2^)**	**Shear stress corresponding to *f*_1_, τ_1 _10^3 ^(N/m^2^)**	**Shear stress corresponding to *f*_2_, τ_1 _10^3 ^(N/m^2^)**
0.2	123.7	0.12	11.8
0.5	77.9	0.31	18.4
1	59.1	0.62	28.3
5	27.8	3.12	64

Let me determine the volume fraction of rouleaux in *f*_2_. It is known that in the absence of blood motion, when the yield velocity equals zero, erythrocytes form rouleaux [29]. First, let me consider the process of particle precipitation in a fluid. The change in dimensionless velocity (*β*) of the precipitation of particles depends on the volume fraction of *f*_2 _(where *β *is the relationship of the velocity of group precipitation to the velocity of a single precipitation). The calculations have been made by other authors, who have proved that the relative velocity can be determined by the formula due to A. D. Maude [32]:

*β *= (1-*f*_2_)^*α*/*m*^

Where *α*/m depends on the Reynolds number; if the Reynolds number equals zero then *α*/m equals 5; if the Reynolds number ranges from 10 to 100 then *α*/m ranges from 4 to 3.5, or:

*μ*_*m *_= (1+*cf*_2_)(1+2.5 *f*_2 _+ 10.05 *f*_2_)

or

β=−cf2+[c2(1−f12)+f13]12
 MathType@MTEF@5@5@+=feaafiart1ev1aaatCvAUfKttLearuWrP9MDH5MBPbIqV92AaeXatLxBI9gBaebbnrfifHhDYfgasaacH8akY=wiFfYdH8Gipec8Eeeu0xXdbba9frFj0=OqFfea0dXdd9vqai=hGuQ8kuc9pgc9s8qqaq=dirpe0xb9q8qiLsFr0=vr0=vr0dc8meaabaqaciaacaGaaeqabaqabeGadaaakeaaiiGacqWFYoGycqGH9aqpcqGHsislcqWGJbWycqWGMbGzdaWgaaWcbaGaeGOmaidabeaakiabgUcaRmaadmGabaGaem4yam2aaWbaaSqabeaacqaIYaGmaaGcdaqadiqaaiabigdaXiabgkHiTiabdAgaMnaaDaaaleaacqaIXaqmaeaacqaIYaGmaaaakiaawIcacaGLPaaacqGHRaWkcqWGMbGzdaqhaaWcbaGaeGymaedabaGaeG4mamdaaaGccaGLBbGaayzxaaWaaWbaaSqabeaadaWccaqaaiabigdaXaqaaiabikdaYaaaaaaaaa@46AA@

If one considers the sedimentation of a particle in a suspension with viscosity *μ*_*m *_and density *ρ*_*m*_, then the equilibrium equation can be expressed as [31]:

f2ρ2ig−f2ρmg+92f2μma−2(V1−V2)=10     (13)
 MathType@MTEF@5@5@+=feaafiart1ev1aaatCvAUfKttLearuWrP9MDH5MBPbIqV92AaeXatLxBI9gBaebbnrfifHhDYfgasaacH8akY=wiFfYdH8Gipec8Eeeu0xXdbba9frFj0=OqFfea0dXdd9vqai=hGuQ8kuc9pgc9s8qqaq=dirpe0xb9q8qiLsFr0=vr0=vr0dc8meaabaqaciaacaGaaeqabaqabeGadaaakeaacqWGMbGzdaWgaaWcbaGaeGOmaidabeaaiiGakiab=f8aYnaaBaaaleaacqaIYaGmcqWGPbqAaeqaaOGaem4zaCMaeyOeI0IaemOzay2aaSbaaSqaaiabikdaYaqabaGccqWFbpGCdaWgaaWcbaGaemyBa0gabeaakiabdEgaNjabgUcaRmaalaaabaGaeGyoaKdabaGaeGOmaidaaiabdAgaMnaaBaaaleaacqaIYaGmaeqaaOGae8hVd02aaSbaaSqaaiabd2gaTbqabaGccqWGHbqydaahaaWcbeqaaiabgkHiTiabikdaYaaakmaabmGabaGaemOvay1aaSbaaSqaaiabigdaXaqabaGccqGHsislcqWGwbGvdaWgaaWcbaGaeGOmaidabeaaaOGaayjkaiaawMcaaiabg2da9iabigdaXiabicdaWiaaxMaacaWLjaWaaeWaceaacqaIXaqmcqaIZaWmaiaawIcacaGLPaaaaaa@57B2@

Vc=29(ρ2−ρ1)gμ1a2     (14)
 MathType@MTEF@5@5@+=feaafiart1ev1aaatCvAUfKttLearuWrP9MDH5MBPbIqV92AaeXatLxBI9gBaebbnrfifHhDYfgasaacH8akY=wiFfYdH8Gipec8Eeeu0xXdbba9frFj0=OqFfea0dXdd9vqai=hGuQ8kuc9pgc9s8qqaq=dirpe0xb9q8qiLsFr0=vr0=vr0dc8meaabaqaciaacaGaaeqabaqabeGadaaakeaacqWGwbGvdaWgaaWcbaGaem4yamgabeaakiabg2da9maalaaabaGaeGOmaidabaGaeGyoaKdaamaalaaabaWaaeWaceaaiiGacqWFbpGCdaWgaaWcbaGaeGOmaidabeaakiabgkHiTiab=f8aYnaaBaaaleaacqaIXaqmaeqaaaGccaGLOaGaayzkaaGaem4zaCgabaGae8hVd02aaSbaaSqaaiabigdaXaqabaaaaOGaemyyae2aaSbaaSqaaiabikdaYaqabaGccaWLjaGaaCzcamaabmGabaGaeGymaeJaeGinaqdacaGLOaGaayzkaaaaaa@461C@

*ρ*_*m *_= *f*_1_*ρ*_1*i *_+ *f*_2_*ρ*_2*i *_    (15)

Using equations (13), (14) and (15) and the condition *V*_1 _= 0 (velocity of the *f*_1 _phase) it follows that:

μmμ1=f1VcV2
 MathType@MTEF@5@5@+=feaafiart1ev1aaatCvAUfKttLearuWrP9MDH5MBPbIqV92AaeXatLxBI9gBaebbnrfifHhDYfgasaacH8akY=wiFfYdH8Gipec8Eeeu0xXdbba9frFj0=OqFfea0dXdd9vqai=hGuQ8kuc9pgc9s8qqaq=dirpe0xb9q8qiLsFr0=vr0=vr0dc8meaabaqaciaacaGaaeqabaqabeGadaaakeaadaWcaaqaaGGaciab=X7aTnaaBaaaleaacqWGTbqBaeqaaaGcbaGae8hVd02aaSbaaSqaaiabigdaXaqabaaaaOGaeyypa0ZaaSaaaeaacqWGMbGzdaWgaaWcbaGaeGymaedabeaakiabdAfawnaaBaaaleaacqWGJbWyaeqaaaGcbaGaemOvay1aaSbaaSqaaiabikdaYaqabaaaaaaa@3B87@

where *α *is the diameter, *μ *is viscosity and *ρ *is the density of the mixture

Taking into account *β*, I deduce the following:

μmμ1=f1/((c2(1−f1)2+f13)−cf2)
 MathType@MTEF@5@5@+=feaafiart1ev1aaatCvAUfKttLearuWrP9MDH5MBPbIqV92AaeXatLxBI9gBaebbnrfifHhDYfgasaacH8akY=wiFfYdH8Gipec8Eeeu0xXdbba9frFj0=OqFfea0dXdd9vqai=hGuQ8kuc9pgc9s8qqaq=dirpe0xb9q8qiLsFr0=vr0=vr0dc8meaabaqaciaacaGaaeqabaqabeGadaaakeaadaWcaaqaaGGaciab=X7aTnaaBaaaleaacqWGTbqBaeqaaaGcbaGae8hVd02aaSbaaSqaaiabigdaXaqabaaaaOGaeyypa0JaemOzay2aaSbaaSqaaiabigdaXaqabaGccqGGVaWlcqGGOaakdaGcaaqaaiabcIcaOiabdogaJnaaCaaaleqabaGaeGOmaidaaOGaeiikaGIaeGymaeJaeyOeI0IaemOzay2aaSbaaSqaaiabigdaXaqabaGccqGGPaqkdaahaaWcbeqaaiabikdaYaaakiabgUcaRiabdAgaMnaaDaaaleaacqaIXaqmaeaacqaIZaWmaaGccqGGPaqkaSqabaGccqGHsislcqWGJbWycqWGMbGzdaWgaaWcbaGaeGOmaidabeaakiabcMcaPaaa@4D8F@

Taking into account that rouleaux sediment in a medium that contains plasma, erythrocytes and small number of rouleaux (i.e. rouleaux are almost destroyed when the yield velocity exceeds 500 c^-1 ^[21]), *μ*_1 _can be calculated according to Einstein formula:

μmμ1=1+cf2
 MathType@MTEF@5@5@+=feaafiart1ev1aaatCvAUfKttLearuWrP9MDH5MBPbIqV92AaeXatLxBI9gBaebbnrfifHhDYfgasaacH8akY=wiFfYdH8Gipec8Eeeu0xXdbba9frFj0=OqFfea0dXdd9vqai=hGuQ8kuc9pgc9s8qqaq=dirpe0xb9q8qiLsFr0=vr0=vr0dc8meaabaqaciaacaGaaeqabaqabeGadaaakeaadaWcaaqaaGGaciab=X7aTnaaBaaaleaacqWGTbqBaeqaaaGcbaGae8hVd02aaSbaaSqaaiabigdaXaqabaaaaOGaeyypa0JaeGymaeJaey4kaSIaem4yamMaemOzay2aaSbaaSqaaiabikdaYaqabaaaaa@3983@

My previous work [21] shows the relationship to blood viscosity when the yield velocity is zero. This allows me now to calculate the concentrations of rouleaux inside the branching part of the vessel on both the external and internal parts of the vessel wall.

Tables 7, 8 and 9 show that if the yield velocity slightly increases then the shear stress of *f*_2 _increases sharply, and then it will result in the destruction of rouleaux (the yield velocity is 5). The viscosity of *f*_2 _considerably exceeds the viscosity of *f*_1_; thus, an increase of the viscosity of a mixture results in decreased yield velocity. Furthermore, the increase in yield velocity results in a decrease of viscosity of *f*_2_, although the shear stress of *f*_2 _increases owing to the high concentration of erythrocytes. From tables 7, 8 and 9 one can observe that the major stress created by blood flow can be expressed as the shear stress of *f*_2_.

According to tables 7, 8, 9, the shear stress of *f*_2 _is the major factor. The shear stress of this second phase depends on both erythrocytes and rouleaux [21]. The concentration of rouleaux can be calculated taking into account the shear stress and viscosity of the mixture. Tables 10, 11 and 12 show the dependence of rouleau concentration on erythrocyte concentration.

**Table 10:** The dependence of volume fraction of rouleaux from concentration of erythrocytes (28%).

**Yield velocity (m^-1^)**	**Shear stress of a mixture, τ 10^3 ^(N/m^2^), **[29]	**Viscosity of a mixture, (mNcm^-2^)**	**Volume fraction of rouleaux**
0.2	2.4	12	0.17
0.5	5.5	11	0.12
1	8	8	0.048
5	25	5	0
10	40	4	0

**Table 11:** The dependence of volume fraction of rouleaux from concentration of erythrocytes (35.9%).

**Yield velocity, (m^-1^)**	**Shear stress of a mixture, τ 10^3 ^(N/m^2^), **[29]	**Viscosity of a mixture, (mNcm^-2^)**	**Volume fraction of rouleaux**
0.2	5.8	29	0.59
0.5	9	18	0.36
1	14	14	0.24
5	40	8	0.048
10	70	7	0

**Table 12:** The dependence of volume fraction of rouleaux from concentration of erythrocytes (48%).

**Yield velocity, (m^-1^)**	**Shear stress of a mixture, τ 10^3 ^(N/m^2^), **[29]	**Viscosity of a mixture, (mNcm^-2^)**	**Volume fraction of rouleaux**
0.2	12	60	0.87
0.5	19	38	0.61
1	29	29	0.36
5	70	14	0.24
10	110	11	0.12

Table 10, 11, 12 show that the volume fraction of rouleaux depends to a greater extent on the concentration of erythrocytes (which is expressed as the viscosity of the mixture) than on the shear stress. This might be explained by the increase in rouleaux formation when concentration of erythrocytes is high.

Therefore, very importantly, for a specific part of the external wall of the vessel, a blood viscosity of 9 mNcm^-2 ^and a volume fraction of rouleaux = 0.044 may appear only when the concentration of erythrocytes is relatively high (48% or more) and when the yield velocity equals 50 m^-1^. Thus, only very specific conditions result in the possible formation of rouleaux on the external wall of the vessel.

### C. Internal wall

Let me consider the shear stress on the internal wall, where such stress is usually considered to be low [33]. I take account of the fact that, with the division of flow, redistribution of velocity occurs, which may result in a further 2-3-fold decrease of velocity on the internal wall [29]. The shear stress acting on the internal wall can be calculated according to the formula:

τ=λρu28
 MathType@MTEF@5@5@+=feaafiart1ev1aaatCvAUfKttLearuWrP9MDH5MBPbIqV92AaeXatLxBI9gBaebbnrfifHhDYfgasaacH8akY=wiFfYdH8Gipec8Eeeu0xXdbba9frFj0=OqFfea0dXdd9vqai=hGuQ8kuc9pgc9s8qqaq=dirpe0xb9q8qiLsFr0=vr0=vr0dc8meaabaqaciaacaGaaeqabaqabeGadaaakeaaiiGacqWFepaDcqGH9aqpdaWcaaqaaiab=T7aSjab=f8aYjabdwha1naaCaaaleqabaGaeGOmaidaaaGcbaGaeGioaGdaaaaa@3692@

where *λ *= 0.06 [28]

Tables 13, 14, 15, 16, 17 show that rouleaux are more likely to form on the internal wall of the vessel than on the external one. Blunt trauma can lead to conditions of short-term boundary layer separation on the internal wall of the vessel where rouleaux can become attached [21,27,29]. In addition, as it can be seen in Tables 13, 14, 15, 16, 17 that the higher the initial concentration of erythrocytes, the more rouleaux can be formed on the internal wall of the vessel. Therefore, the smaller the yield velocity, the more rouleaux may form, and these can stick to the internal wall of the vessel. Caro and colleagues [29] conducted an experiment using actual erythrocytes and microspheres made from polyvinyl latex: it was shown that the coefficient of difference changed from 3 10^-8 ^cm^2^c^-1 ^to 1.5 10^-7^cm^2^c^-1^, which significantly exceeded the coefficient of difference for Brownian movement of particles (approximately 4 10^-10 ^cm^2^c^-1^).

**Table 13:** The dependence of rouleaux formation on the concentration of erythrocytes (28%) on the inner wall of the branching part of the vessel.

**Yield velocity, (m^-1^)**	**Shear stress of a mixture, τ 10^3 ^(N/m^2^), **[29]	**Viscosity of a mixture, (mNcm^-2^)**	**Volume fraction of rouleaux**
0.2	0.003	11	0.12
0.5	0.018	7	0.03
1	0.075	4	0.
5	0.3	4	0
10	0.5	5	0

**Table 14:** The dependence of rouleaux formation on the concentration of erythrocytes (35.9%) on the inner wall of the branching part of the vessel.

**Yield velocity, (m^-1^)**	**Shear stress of a mixture, τ 10^3 ^(N/m^2^), **[29]	**Viscosity of a mixture, (mNcm^-2^)**	**Volume fraction of rouleaux**
0.1	0.003	31	0.44
2	0.018	9	0.05
11	0.075	7	0.03
70	0.3	4.3	0
10	0.09	9	0

**Table 15:** The dependence of rouleaux formation on the concentration of erythrocytes (48%) on the inner wall of the branching part of the vessel.

**Yield velocity, (m^-1^)**	**Shear stress of a mixture, τ 10^3 ^(N/m^2^), **[29]	**Viscosity of a mixture, (mNcm^-2^)**	**Volume fraction of rouleaux**
0.05	0.003	60	0.87
0.5	0.018	38	0.61
5	0.075	14	0.24
30	0.3	10	0.11
10	0.15	15	0.24

**Table 16:** The dependence of rouleaux formation on the concentration of erythrocytes (58.9%) on the inner wall of the branching part of the vessel.

**Yield velocity, (m^-1^)**	**Shear stress of a mixture, τ 10^3 ^(N/m^2^), **[29]	**Viscosity of a mixture, (mNcm^-2^)**	**Volume fraction of rouleaux**
1	0.052	21	0.2
5	0.105	44	0.51
10	0.3	18	0

**Table 17:** The dependence of rouleaux formation on the concentration of erythrocytes (67.4%) on the inner wall of the branching part of the vessel.

**Yield velocity, (m^-1^)**	**Shear stress of a mixture, τ 10^3 ^(N/m^2^), **[29]	**Viscosity of a mixture, (mNcm^-2^)**	**Volume fraction of rouleaux**
10	0.3	22.7	0
100	0.3	11.1	0.12
300	0.3	9.8	0.11

## Discussion

As mentioned earlier, aortic injury carries a significant mortality with on-scene death occurring in more than 80% of individuals sustaining this injury [2,7]. In the study conducted by Fabian et al. [34], 274 blunt aortic injury cases were studied over a period of 2.5 years. The lethality was 31%, with 63% of deaths due to aortic rupture. Fabian et al. [34] concluded that aortic rupture remains a major problem. The high mortality associated with delayed diagnosis has justified the search for potential risk factors and diagnostic tests [23].

Attempts to identify such risk factors have been made in several research studies. Several risk factors were studied by Kram and colleagues [35]. Pelvic fracture, myocardial contusion, intra-abdominal injury and hypotension were found to be significantly associated with aortic injury in their study [35]. In addition to pelvic trauma, Blackmore and colleagues [36] identified other significant predictors for aortic injury including lack of occupant restraint (seat belt) and presence of head injury or pneumothorax. Despite a relatively small sample size, Horton and colleagues [37] found that a deep intrusion (more than 15 inches) near impact and high Delta V contribute significantly to the risk of thoracic aortic injury. At the same time, airbag and seatbelt use were found to have no effect on the incidence of thoracic aortic tear. Finally, a sudden, violent deceleration was found to be important in the incidence of aortic trauma [8,38].

Although aortic injury is uncommon, injury to the aortic isthmus is the most frequent presentation, particularly when associated with widened mediumstinum and blunt chest trauma [39]. In addition, there is always a better potential for survival when the diagnosis for aortic root injury is made early. Future research should identify a set of the most important risk factors, which will help physicians to prevent mortality from aortic injury in emergency room settings.

This study is the first to attempt to identify the "internal" or "intrinsic" risk factors that may predispose an individual to aortic rupture. This study has determined that the shear stresses caused by plasma and erythrocytes differ significantly: the shear stress caused by erythrocytes is much higher than the stress created by plasma. Therefore, the impact of the shear stress caused by the second phase (erythrocytes) may be more significant than the impact of the first phase (plasma).

As noted in previous research, blunt injury to the vascular wall may result in the formation of rouleaux [21]. Therefore, the impact of the shear stress caused by the second phase may become particular prominent in trauma patients. However, the impact of the shear stress created by rouleaux is even greater then that of the shear stress from erythrocytes because of the size differences. Therefore, in trauma patients, the risk of aortic rupture is related to geometric peculiarities such as geometry of the vessel (the Dean number) and the extent of rouleaux formation. This may provide insights into the delayed rupture of the branching part of the vessels in the posttraumatic period (Table 18). According to this research, rouleaux formation due to trauma may lead to increased shear stress. Even if this increase is only 1.5-2-fold in a straight vessel, it is 4 times greater on the internal part of the vessel. Therefore, in some parts of the vascular system, the shear stress may increase up to 120 – 140 N/m^2 ^(a shear stress of 40 N/m^2 ^or more can damage the endothelium of the vessel, as mentioned earlier).

**Table 18:** Summary of some case reports that provide information on time interval between trauma (motor vehicle crash) and acute myocardial infarction.

**Author**	**Age**	**Mechanism of injury**	**Time interval between trauma and acute myocardial infarction**
Boland et al. [14]	32	Motor vehicle crash	4 days
Candell et al. [19]	38	Motor vehicle crash	24 hours
Foussas et al. [47]	26	Motor vehicle crash	17 days
Lee et al. [48]	54	Motor vehicle crash	3 days
Lehmus et al. [49]	62	Motor vehicle crash	1.5 hours
Oliva et al. [50]	44	Motor vehicle crash	24 hours
Vlay et al. [51]	25	Motor vehicle crash	5 days

In general, substantial variations in the geometric parameters of human arteries have been recognized as knowledge of the geometric peculiarities of coronary vessels has advanced, perhaps because of their clinical significance [40-42]. In a study by Hutchins et al. [41], the range of angles in 56 coronary artery branches was shown to vary from 32 to 124 degrees. Both in vitro [43] and in vivo [44] studies have revealed substantial variation in arterial geometry at human aortic bifurcations. Arterial geometry has been suggested to play the role in hemodynamics and atherosclerosis [45]. Friedman et al. [46] suggested that various geometrical configurations of the vessel may result in different distributions of mechanical stress in the wall.

Our experience shows that various types of trauma may result in such serious outcomes as acute myocardial infarction [20,21,49,51,52]. Recognition of the fact that certain geometrical peculiarities in coronary arteries may predispose them to delayed rupture trauma may become important not only for identifying screening and treatment procedures to prevent further myocardial damage from trauma, but also to prevent further complications such as ventricular fibrillation. This is particularly important since acute myocardial infarction resulting from trauma has been shown to occur several hours or days after the trauma [19,49-52] (Table 18).

On the other hand, in some studies, pelvic and intra-abdominal injuries have been shown to be significantly associated with aortic injury. At the same time, there is no consensus about the capacity of seatbelt use to protect from such types of injury. The knowledge that certain geometric and rheological peculiarities may predispose a particular person to the impact of traumatic injury may help us to identify the forces affecting the abdominal and pelvic areas and, therefore, to maximize the protective effects of seat belts and other safety devices.

## Conclusion

The results of this research take into account certain geometrical peculiarities of the branching part of the vessel. The mathematical model created in this study will improve our understanding of the complex mechanism of blunt injury to the vascular wall and, therefore, conditions such as aortic rupture and traumatic acute myocardial infarction.

## Competing interests

The author(s) declare that they have no competing interests.
